# A *Drosophila* Model of Essential Tremor

**DOI:** 10.1038/s41598-018-25949-w

**Published:** 2018-05-16

**Authors:** Philip Smith, Ronald Arias, Shilpa Sonti, Zagaa Odgerel, Ismael Santa-Maria, Brian D. McCabe, Krasimira Tsaneva-Atanasova, Elan D. Louis, James J. L. Hodge, Lorraine N. Clark

**Affiliations:** 1 School of Physiology, Pharmacology and Neuroscience, University of Bristol, University Walk, Bristol, BS8 1TD UK; 2 Department of Pathology and Cell Biology, College of Physicians and Surgeons, Columbia University, New York, NY 10032 USA; 3 Brain Mind Institute, Swiss Federal Institute of Technology (EPFL), Lausanne, Switzerland; 4 Department of Mathematics and Living Systems Institute, University of Exeter, Stocker Road, Exeter, EX4 4QD UK; 5 EPSRC Centre for Predictive Modelling in Healthcare, University of Exeter, Exeter, EX4 4QJ UK; 6 Department of Neurology, Yale School of Medicine, Yale University, New Haven, CT 06520 USA; 7 Center for Neuroepidemiology and Clinical Neurological Research, Yale School of Medicine, Yale University, New Haven, CT 06520 USA; 8 Department of Chronic Disease Epidemiology, Yale School of Public Health, New Haven, CT 06520 USA; 9 Taub Institute for Research on Alzheimer’s Disease and the Aging Brain, College of Physicians and Surgeons, Columbia University, New York, NY 10032 USA

## Abstract

Essential Tremor (ET) is one of the most common neurological diseases, with an estimated 7 million affected individuals in the US; the pathophysiology of the disorder is poorly understood. Recently, we identified a mutation (*KCNS2* (*Kv9.2*), c.1137 T > A, p.(D379E) in an electrically silent voltage-gated K^+^ channel α-subunit, *Kv9.2*, in a family with ET, that modulates the activity of Kv2 channels. We have produced transgenic *Drosophila* lines that express either the human wild type Kv9.2 (hKv9.2) or the ET causing mutant Kv9.2 (hKv9.2-D379E) subunit in all neurons. We show that the hKv9.2 subunit modulates activity of endogenous *Drosophila* K^+^ channel Shab. The mutant hKv9.2-D379E subunit showed significantly higher levels of Shab inactivation and a higher frequency of spontaneous firing rate consistent with neuronal hyperexcitibility. We also observed behavioral manifestations of nervous system dysfunction including effects on night time activity and sleep. This functional data further supports the pathogenicity of the *KCNS2* (p.D379E) mutation, consistent with our prior observations including co-segregation with ET in a family, a likely pathogenic change in the channel pore domain and absence from population databases. The *Drosophila* hKv9.2 transgenic model recapitulates several features of ET and may be employed to advance our understanding of ET disease pathogenesis.

## Introduction

Recent studies suggest that ET could be a neurodegenerative disorder, primarily affecting the cerebellar system, accompanied by changes in the Purkinje cell population and altered (i.e. reduced) brain GABA tone^1^; however, this is far from established. Recently, we identified a mutation in the *KCNS2* gene (*Kv9.2*, c.1137 T > A, p.(D379E), encoding an electrically silent voltage-gated K^+^ channel α-subunit, Kv9.2, in a single family with early-onset ET^2^. The *KCNS2* mutation cosegregated with ET in this family and was present in all four affected individuals and absent in an unaffected family member.

*KCNS2* encodes an electrically silent voltage-gated K^+^ channel α-subunit, Kv9.2, that is highly and selectively expressed in the brain and modulates the activity of the Kv2.1 and Kv2.2 channels by heteromulterization. A similar localization of expression of Kv9.2 with Kv2.1 and Kv2.2 has been observed in the Purkinje and granular cells in mouse cerebellum^3^.

In *Drosophila*, voltage gated potassium ion (Kv) channels play a major role in regulating membrane excitability and synaptic transmission in many central neurons and at the neuromuscular junction (NMJ)^4^. Thus, *Drosophila* is a useful model system for investigating ion channel kinetics and their impact on neuronal firing properties. A direct ortholog of Kv9.2 is absent from *Drosophila* with the closest homologue to Kv9.2 in *Drosophila* being Shab (42% amino acid identity and 63% amino acid similarity). The *Drosophila* genome encodes four prototypic members of the Kv channel gene family, namely, *Shaker (Kv1)*, *Shab (Kv2)*, *Shaw (Kv3)*, and *Shal (Kv4)*^5^. In *Drosophila* neurons *Shab* encodes the majority of delayed rectifier current (I_k_) and is a member of the Kv2 subfamily^5,6^. *Drosophila* Shab I_k_ is important in the regulation of high frequency repetitive synaptic activity and removal of Shab I_k_ dramatically increases NMJ transmission (up to 10-fold gain) during repetitive nerve stimulation^4^. The kinetics of the Shab channel are reported to be comparable to the classical (as described by Hodgkin–Huxley) delayed rectifier K^+^ channel^6^.

Abnormal motor behavior and leg shaking (with or without anesthesia) has been described in *Shab* mutant files^4^. Many neurological disorders are associated with altered activity, function or expression of K^+^ channels^7^ and mutations in these channels can cause cerebellar dysfunction and ataxia. Notably, a tremor phenotype has been described in patients with mutations in *KCNA1* (Kv1.1; EA; OMIM 160120)^8^ and missense dominant negative mutations in *KCNC3* (Kv3.3) are associated with hyperexcitability, cerebellar neurodegeneration and subsequent movement defects including spinocerebellar ataxia (SCA13; OMIM 605259)^9^. Kv3.1 and Kv3.3 mutant mice also display severe motor deficits, including tremor, myoclonus, and ataxic gait and behavioral alterations that include constitutive hyperactivity and sleep loss^10–12^. In addition to inherited channelopathies, several neurodegenerative disorders including Alzheimer’s disease, Parkinson’s disease, Huntington’s disease, amyotrophic lateral sclerosis, and SCAs exhibit altered properties of diverse K^+^ channels characterized by protein aggregate induced hyperexcitability (Kumar *et al*.^13^).

Currently, it is unclear how the mutation that we identified in Kv9.2 (p.D379E) could lead to an ET phenotype. However, the dominant inheritance pattern suggests a gain-of-function or dominant negative mechanism. To study the disease mechanism we have produced transgenic *Drosophila* lines that express either the wild type human Kv9.2 (hKv9.2) subunit or the ET causing mutant human Kv9.2 (hKv9.2-D379E) subunit in all neurons. Here, we show that the hKv9.2 subunit can modulate the channel activity of endogenous *Drosophila* Shab (Kv2), describe the behavioral manifestations of nervous system dysfunction, and the effects of hKv9.2 and hKv9.2-D379E on adult neuron activity.

## Results

To study the disease mechanism of the Kv9.2 (p.D379E) mutation that we identified in an ET family and advance our understanding of disease pathogenesis, we have produced transgenic *Drosophila* lines that express the wild type hKv9.2 or ET causing mutant hKv9.2-D379E subunit.

Pan-neural expression of the wild type hKv9.2 or mutant hKv9.2-D379E subunit resulted in no apparent gross morphological differences. Western blot analysis was used to verify transgenic expression of hKv9.2 and hKv9.2-D379E in *Drosophila*. Using a hKv9.2 immunogen corresponding to amino acids 175–470, we detected a 54.2 kDa band of the correct size corresponding to human hKv9.2 (Fig. S1).

### Behavioral Manifestations of Nervous System Dysfunction

**Figure 1:**
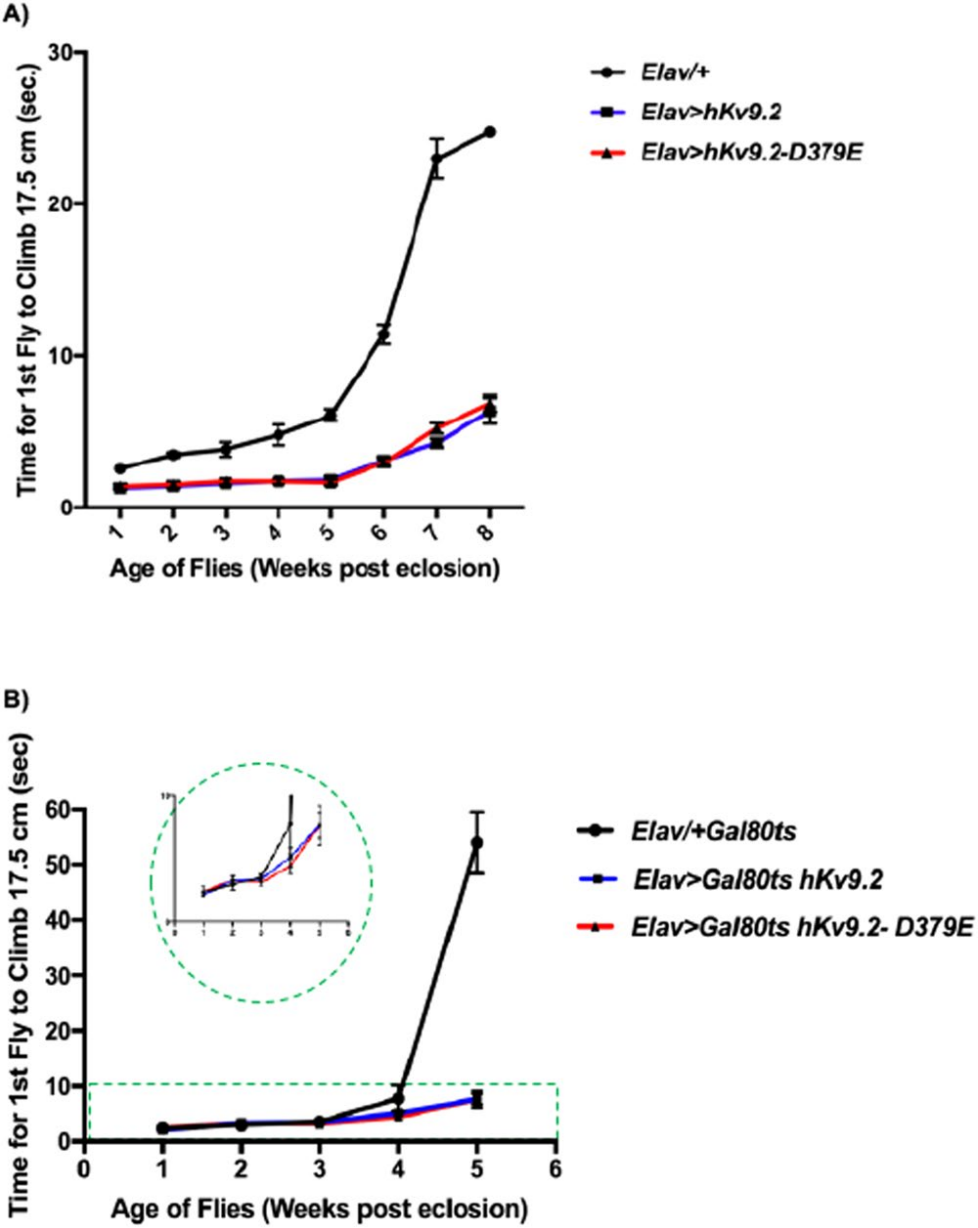
Climbing Response During Lifespan. The climbing index was assessed as the time taken for the first fly to climb 17.5 cm. (**A**) Expression of hKv9.2 and hKv9.2-D379E channels throughout neuronal development and effects on climbing response during lifespan. The mean climbing index ± SEM as a function of age is shown for *Elav*/+, *Elav* > *hKv9.2* and *Elav* > *hKv9.2-D379E*. Each point represents the mean of 10 flies except for: Week 1, *Elav* > *hKv9.2* (n = 9); Weeks 1–8, *Elav* > *hKv9.2-D379E* (n = 11); Week 6, *Elav*/+ (n = 7); Week 8, *Elav*/+ (n = 1). Flies expressing either *Elav* > *hKv9.2* and *Elav* > *hKv9.2-D379E* displayed significantly faster climbing (*p* < 0.0003) throughout lifespan. (**B**) Expression of hKv9.2 and hKv9.2-D379E channels post developmentally in neurons and effects on climbing response during lifespan. The mean climbing index ± SEM as a function of age is shown for *Elav-gal4*/+ *Gal80*^*ts*^, *Elav-gal4* > *Gal80*^*ts*^ *hKv9.2* and *Elav-gal4* > *Gal80*^*ts*^ *hKv9.2-D379E*. Each point represents the mean of 10 or 9 flies. Flies expressing either *Elav* > *hKv9.2* and *Elav* > *hKv9.2-D379E Elav-gal4* > *Gal80*^*ts*^ displayed significantly faster climbing (*p* = 0.04–0.0001; weeks 3–5) during lifespan.

To test the hypothesis that mutations in Kv9.2 cause nervous system dysfunction, we tested the effect of pan-neural expression of hKv9.2-D379E compared to wild type hKv9.2 or controls on climbing response throughout the fly lifespan^14^ (Fig. 1). Flies expressing either wild type or mutant human channels displayed significantly faster climbing (*p* < 0.0003) throughout lifespan (Fig. 1A), consistent with the hKv9.2 channel expressing animals being behaviorally hyperexcitable as previously described for mutants of other Kv channels in locomotor assays^15,16^. Moreover, flies which only expressed the wild type or mutant hKv9.2 channel in adult neurons after development also displayed significantly faster climbing throughout lifespan (*p* = 0.04–0.0001; weeks 3–5) suggesting that the phenotype is not due solely to changes during development (Fig. 1B).

**Figure 2:**
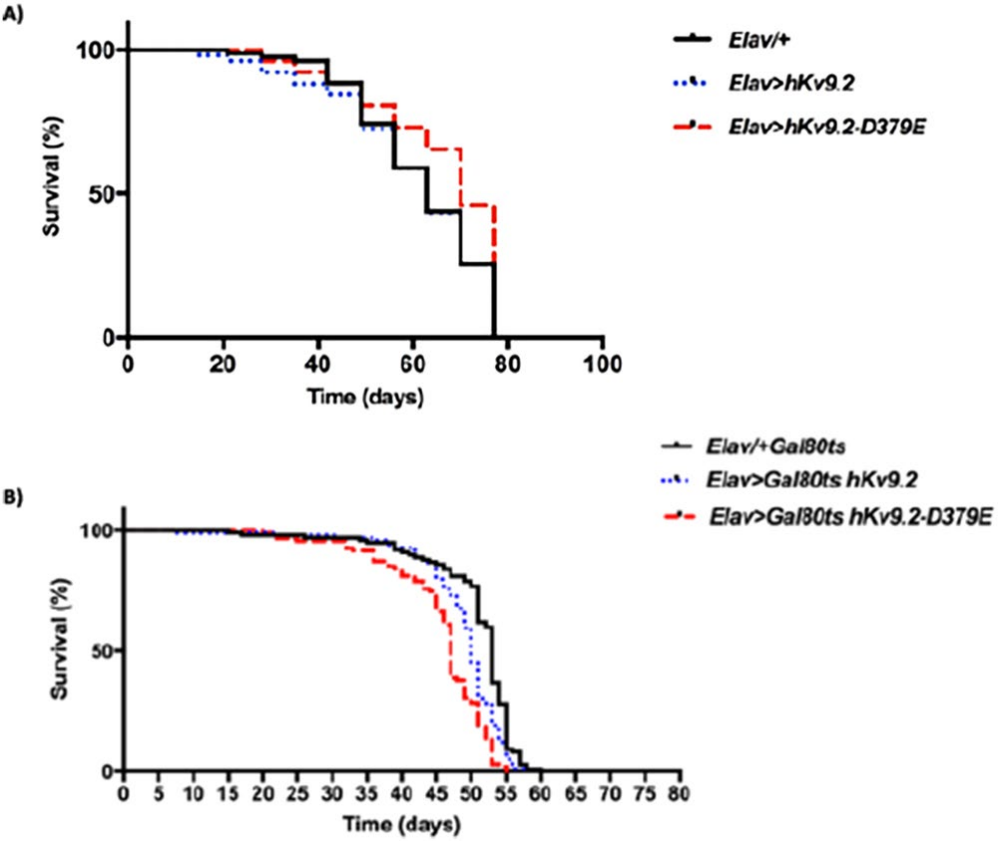
Lifespan Assays in hKv9.2 Transgenic Lines. (**A**) Expression of hKv9.2 and hKv9.2-D379E channels throughout neuronal development and effects on lifespan. A total of 200 virgin flies per line were sex-segregated within 4 h of eclosion and maintained in small laboratory vials (n = 20 per vial) containing fresh food in a low-temperature incubator at 25 °C and 40% humidity on a 12/12 h dark/light cycle. The flies were then transferred to fresh food vials every 3-4 days and mortality recorded. Significant differences were not observed between *Elav*/+ and *Elav* > *hKv9.2* (*p* = 0.8771) or *Elav*/+ and *Elav* > *hKv9.2-D379E* (*p* = 0.0656). (**B**) Expression of hKv9.2 and hKv9.2-D379E channels post developmentally in neurons and effects on lifespan. A total of 100 virgin flies per line were sex-segregated within 4 h of eclosion and age-matched flies were maintained in small laboratory vials (n = 10 per vial) containing fresh food in a high-temperature incubator at 29 °C and 40% humidity on a 12/12 h dark/light cycle. The flies were then transferred to fresh food vials every 2-3 days and mortality recorded. Significant differences were observed between *Elav-gal4*/+*Gal80*^*ts*^ and *Elav-gal4* > *Gal80*^*ts*^ *hKv9.2* (*p* = 0.0012) or *Elav-gal4*/+*Gal80*^*ts*^ and *Elav-gal4 Gal80*^*ts*^ > *hKv9.2-D379E* (*p* = 0.0001).

Because ET is associated with increased mortality^17^ and Kv channel dysfunction in both humans and flies is associated with disease and decreased lifespan (Kumar *et al*.^13^) we performed survival assays. Significant differences in lifespan were not detected between flies expressing hKv9.2 or hKv9.2-D379E channels during development compared to wild type (Fig. 2A). Flies expressing the wild type or mutant hkv9.2 channel post-developmentally in neurons however did display significant differences in lifespan compared to wild type (*Elav-gal4*/+ *Gal80*^*ts*^ and *Elav-gal4* > *Gal80*^*ts*^ *hKv9.2* (*p* = 0.0012) or *Elav-gal4*/+ *Gal80*^*ts*^ and *Elav-gal4* > *Gal80*^*ts*^ *hKv9.2-D379E*. (*p* = 0.0001)). (Fig. 2B). The reduced lifespan observed for flies expressing the hKv9.2 or mutant hKv9.2 channel post-developmentally is consistent with a reduced lifespan observed for other hyperexcitable Kv channel mutants^13^.

Wing posture and motility deficits were also assessed. Normally, flies hold their wings flat and rarely display an elevated or downturned wing posture. However, we observed that approximately 40% of flies expressing hKv9.2 or hKv9.2-D379E pan-neuronally displayed an abnormal wing posture, with bilateral wing elevation, with onset 7–21 days post eclosion (Supplementary Movie 1). This abnormality may be due to muscle and/or neural based effects and a downturned wing phenotype has been described in other *Drosophila* models of neurodegeneration including Huntington’s disease^18^ and Parkinson’s disease^19,20^ and bilateral wing elevation has been described for flies expressing the ion channel gene, *TRPM8*, in adult neurons^21^. Alternatively this effect could also be due to a developmental effect or muscle based hyperexcitability as described in the *Drosophila ether-à-go-go* (*eag*) *Shaker* double mutant^22^. To test whether the abnormal wing posture observed in flies expressing hKv9.2 or hKv9.2-D379E pan-neuronally was due to a developmental effect we also assessed flies expressing the wild type or mutant hKv9.2 channel only in adult neurons. We observed that approximately 13% of flies expressing hKv9.2-D379E in adult flies displayed an abnormal wing posture, with bilateral wing elevation, with onset 6 weeks post eclosion (*p* < 0.0001)(Fig. S2). Abnormal wing posture and motility defects were also observed in flies expressing the wild type hKv9.2 channel in post-developmental neurons (~20% also with onset 6 weeks after eclosion (*p* < 0.0001) (Fig. S2)).

Hyperexcitable mutants such as *Shaker, Shab, Shaw, eag and Hyperkinetic* display abnormal leg shaking and wing scissoring in etherised adults^23–25^. Adult flies expressing hKv9.2 or hKv9.2-D379E pan-neuronally also exhibited leg shaking, abdominal pulsations and body shuddering under ether anesthetization (Supplementary Movie 2). The ether anesthetization induced tremor in these flies is consistent with reports in the literature of excitatory effects of commonly used anesthestics in humans that may manifest as spontaneous movements including tremor, dystonia and myoclonus^26^. Interestingly, a number of observations suggest a link between anesthesia and ET, including an ET kindred with malignant hyperthermia^27^ and a porcine model of malignant hyperthermia manifesting high frequency tremor^28^.

### Expression of either hKv9.2 and hKv9.2-D379E causes an alteration in the kinetics of Shab (Kv2)

**Figure 3:**
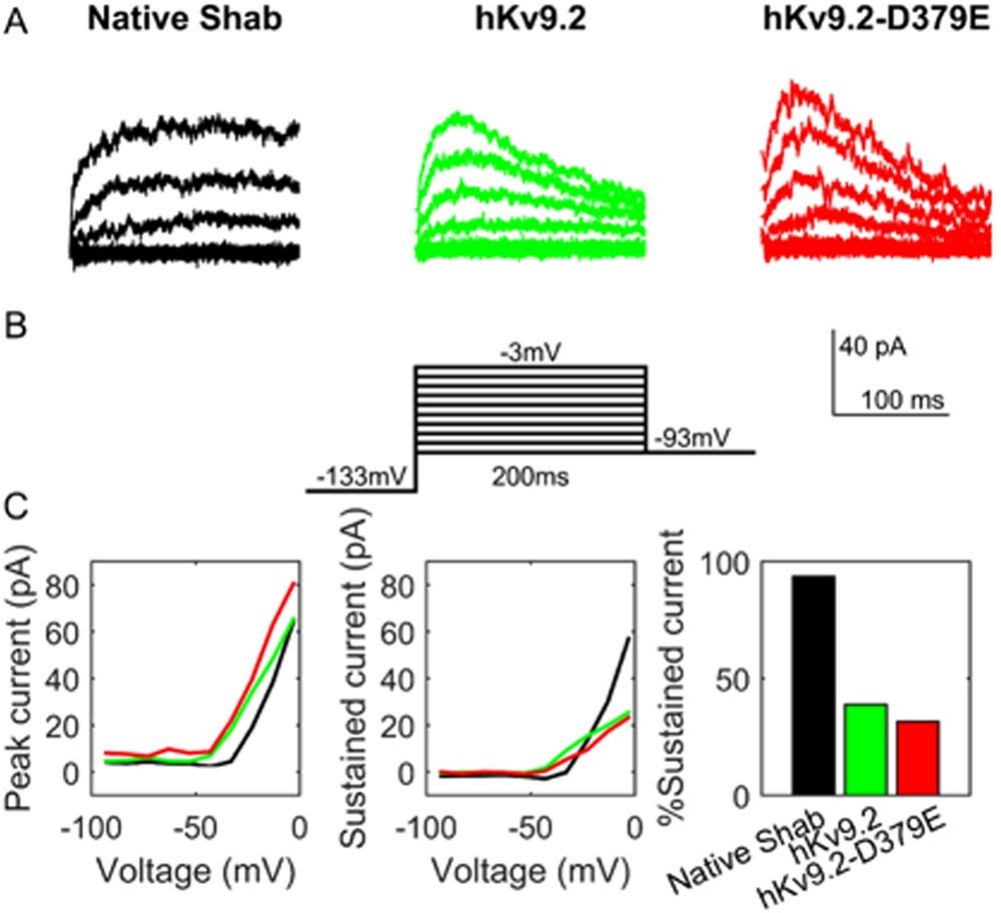
Expression of hKv9.2 or hKv9.2-D379E subunits causes an alteration in the kinetics of the native Shab (Kv2) channels. (**A**) Examples of the current evoked from *Drosophila* Shab when depolarized from −133 mV to between −93 mV and −3 mV in flies expressing only native Shab (left panel), native Shab with the human Kv9.2 (middle panel), or native Shab with hKv9.2-D379E (right panel). The non-inactivating Shab becomes inactivating in the presence of either hKv9.2 subunit. (**B**) Diagram of the voltage step protocol used. (**C**) The I-V relationships for Shab determined from the three given genotypes. The peak current (left panel, n = 3) shows that expression of either Kv9.2 subunit causes a shift in activation to more negative voltages. The sustained current (middle panel, n = 3) shows that, at similar voltages, flies expressing either hKv9.2 subunit show reduced Shab current after 200 ms of depolarization. When the sustained current after 200 ms of depolarization to −3 mV is expressed as a percentage of the peak current (right panel, n = 3) the flies expressing only native Shab show high percentages, indicating very low levels of inactivation. When tested by two-way ANOVA, flies expressing either the wild-type hKv9.2 subunit (*p* < 0.001) or the ET mutant hKv9.2-D379E subunit (*p* < 0.001) were significantly different to flies expressing only native Shab. The mutant hKv9.2-D379E subunit shows much higher levels of inactivation than the wild-type hKv9.2 subunit (*p* = 0.0462).

We determined the effects of hKv9.2 and hKv9.2-D379E on neuron activity because ether anesthetization induced tremor in flies. hKv9.2 and hKv9.2-D379E transgenes were expressed in flies and whole cell voltage clamp recordings were performed on the large lateral neuron ventral (l-LNv) clock neurons, which have been employed as model central neurons for electrophysiolgical measurements in *Drosophila*^29,30^. Using a Shab specific toxin we were able to isolate the Shab current (a voltage-sensitive non-inactivating K^+^ current) in these neurons consistent with previous reports^6^. Expression of either hKv9.2 and hKv9.2-D379E in these neurons caused an alteration in the kinetics of Shab (Kv2) (Fig. 3A). The current evoked from Shab when depolarized from −133 mV to −3 mV showed that the non-inactivating Shab becomes inactivating in the presence of the wild type hKv9.2 subunit and to a greater extent with the mutant hKv9.2-D379E subunit (Fig. 3A). The I-V relationships for Shab in the three given genotypes was also determined (Fig. 3C). The peak current shows that expression of the hKv9.2 subunits cause a shift in activation of Shab to more negative voltages. The sustained current shows that, at similar voltages, flies expressing either hKv9.2 subunit show reduced Shab current after 200 ms of depolarization. When the sustained current after 200 ms of depolarization is expressed as a percentage of the peak current the flies with only native Shab (i.e. controls) show high percentages, indicating very low levels of inactivation (Fig. 3C). Flies expressing either the wild type hKv9.2 subunit *(p* < 0.001) or the mutant hKv9.2-D379E subunit (*p* < 0.001) were significantly different to flies with only endogenous Shab alone (control). Comparing the relative sustained currents (Fig. 3C), the mutant hKv9.2-D379E subunit shows significantly higher levels of inactivation than the wild type hKv9.2 subunit (n = 3, *p* = 0.0438). The observed change in the inactivation kinetics of Shab in the presence of mutant hKv9.2-D379E subunit would be predicted to result in neuronal hyperexcitability.

### Expression of mutant Kv9.2-D379E significantly increases the firing frequency

**Figure 4:**
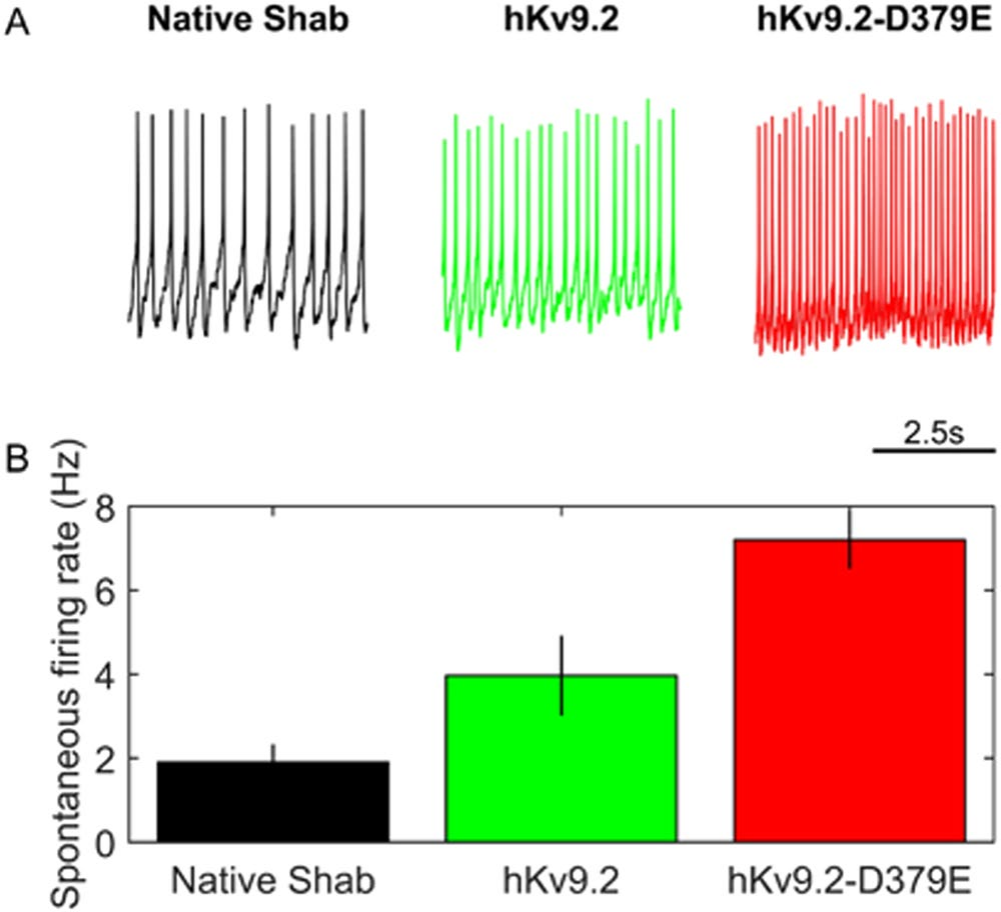
hKv9.2 subunits have significant and different impacts on resulting neuronal activity. (**A**) Representative example of 5 seconds of spontaneous activity in flies between ZT1 and ZT7 (where ZT0 is lights-on and ZT12 is lights-off) expressing only native Shab (left panel), native Shab with hKv9.2 (middle panel), or native Shab with hKv9.2-D379E (right panel). (**B**) The spontaneous firing rate of action potentials in flies expressing either hKv9.2 subunit (data are mean ± S.D., n = 3) are significantly different with the mutant subunit having a higher frequency (t(4) = 5.3806, p = 0.0102).

**Figure 5:**
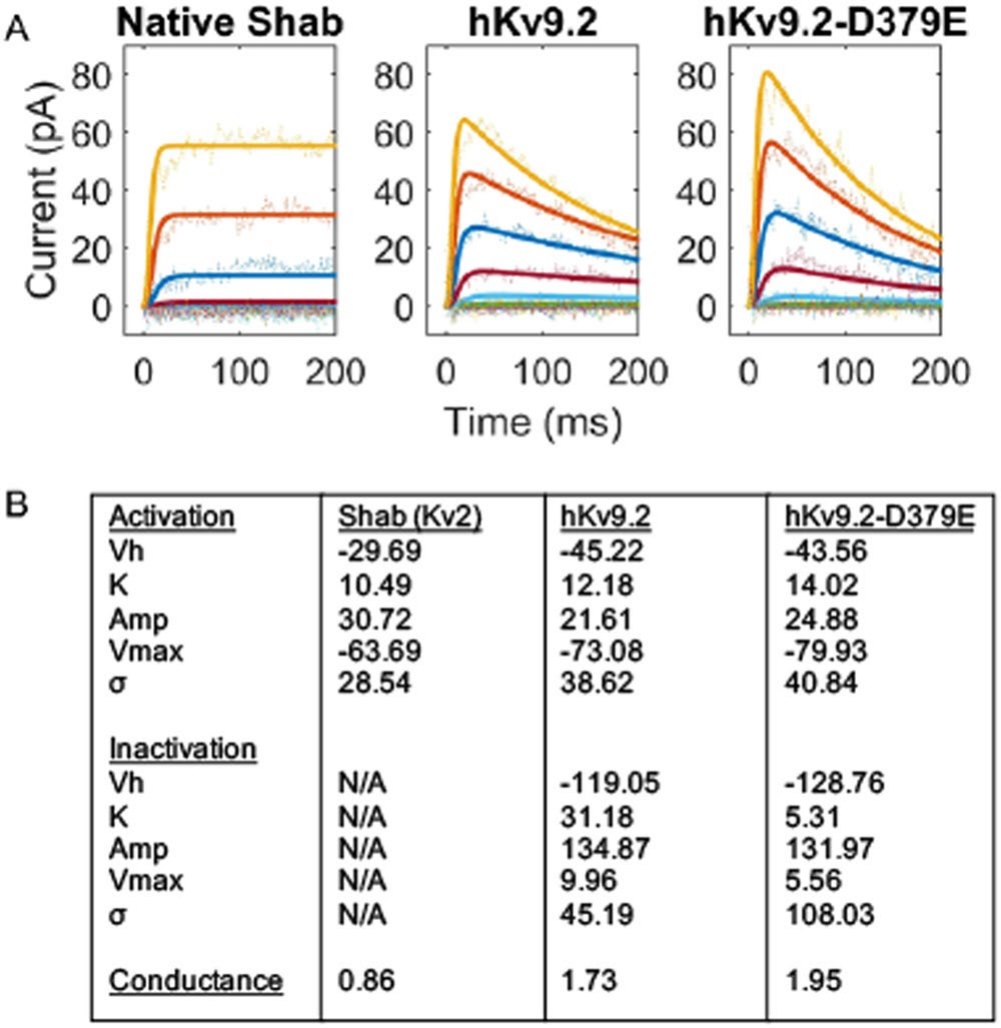
Computational models of Shab ion channel activity describe their differing kinetics. Fitting the electrophysiological data (see Fig. 1) to classical Hodgkin-Huxley equations generated parameters that describe the behaviors (**B**). These descriptions match well with experimental data acquired (**A**). The hKv9.2 subunits activate at more negative values (activation Vh) as seen in the experimental data. The extent of inactivation is higher in the mutant hKv9.2-D379E as evidenced by the lower inactivation K. The speed of inactivation is also higher in the mutant hKv9.2-D379E as shown by the higher inactivation σ.

**Figure 6:**
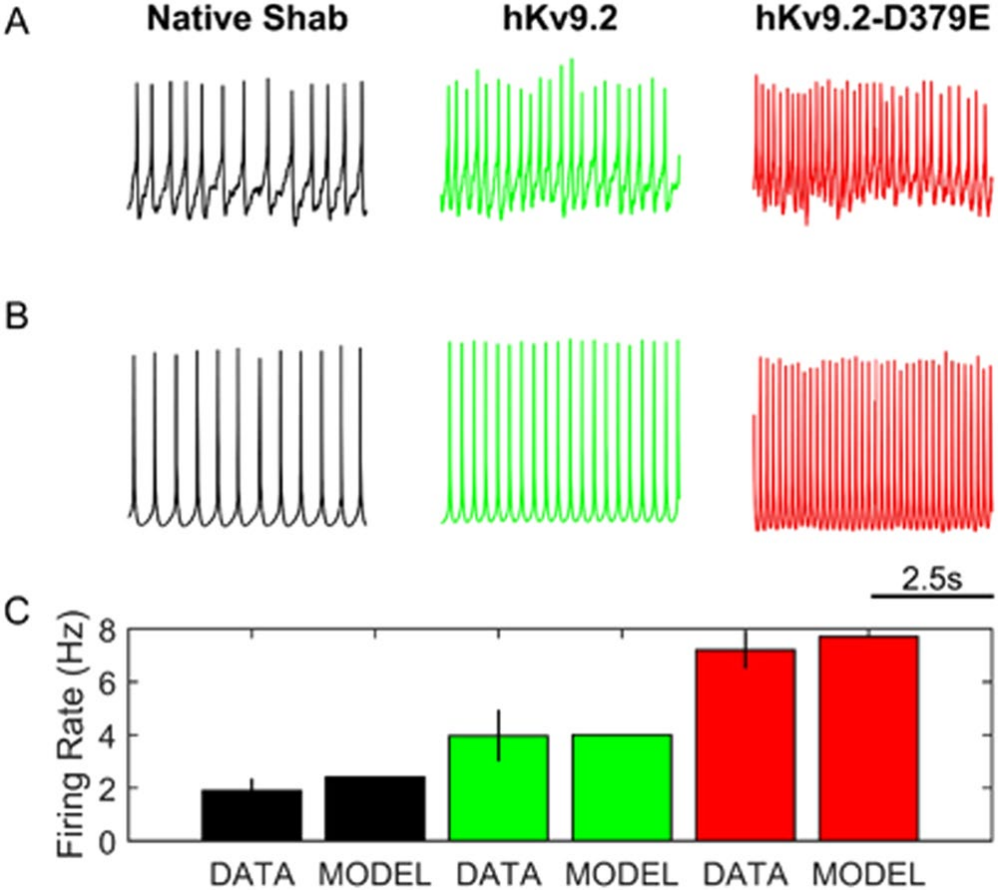
Whole cell computational models using hKv9.2 reflect changes in electrophysiological behavior. (**A**) Representative example of 5 seconds of spontaneous activity in flies expressing only native Shab (left panel), native Shab with hKv9.2 (middle panel), or native Shab with hKv9.2-D379E (right panel). (**B**) Model description of 5 seconds of neuronal activity using only native Shab (left panel), native Shab with hKv9.2 (middle panel), or native Shab with hKv9.2-D379E (right panel) at ZT0 (where ZT0 is lights-on/dawn and ZT12 is lights-off/dusk). (**C**) The model description of the spontaneous firing rate of action potentials in flies expressing either hKv9.2 subunit is not significantly different to the experimental data.

To test whether the hKv9.2-D379E subunit does indeed cause neuronal hyperexcitability, we performed current clamp recordings of clock neurons. We found that expression of hKv9.2-D379E subunit caused a significant increase in firing frequency (a measure of hyperexcitability) compared to expression of the hKv9.2 subunit (Fig. 4). The spontaneous action potential firing rate in flies expressing either hKv9.2 subunit was significantly different with the mutant subunit having a higher frequency (*p* = 0.0102) (Fig. 4A,B). In order to further investigate the complex effects of expression of hKv9.2 and hKv9.2-D379E on Shab currents in *Drosophila* neurons, we developed a biophysical model that incorporated the major Kv currents separately. Major Kv current kinetics were fitted to the voltage-clamp data so that an accurate depiction of the specific effects of expression of hKv9.2 and hKv9.2-D379E subunits on the excitability of the whole-cell model could be determined. In particular, fitting the voltage clamp data (see Fig. 5) to classical Hodgkin-Huxley ionic current equations generated parameters consistent with the behaviors of flies with only native Shab or Shab perturbed by expression of hKv9.2 or hKv9.2-D379E on Shab (Fig. 5A,B). As illustrated in Fig. 6 the parameters obtained from the computational modeling are in concordance with experimental data. The hKv9.2 subunit activates at more negative values (activation Vh) than the native Shab channel as observed in the experimental data. The extent of inactivation is also higher in hKv9.2-D379E in the model than in the wild type hKv9.2 flies as evidenced by the lower inactivation K. The speed of inactivation is also higher in the mutant hKv9.2-D379E as shown by the higher inactivation σ. Importantly, the spontaneous firing rate of action potentials obtained in whole-cell model simulations (i.e. after combining the individual ionic currents in a biophysical model of the electrical activity of the cell), in neurons expressing either the wild type or mutant hKv9.2 subunit agrees very well with the experimental (current clamp) data (Fig. 6).

### Expression of either hKv9.2 and hKv9.2-D379E causes a significant increase in night-time activity and reduction in sleep

Sleep dysfunction, including short duration of sleep, has been reported in patients with ET^31–33^. To determine whether the neuronal hyperexcitability observed in hKv9.2 and hKv9.2-D379E in *Drosophila* effects circadian locomotor rhythms and sleep we characterized their circadian behavior.

**Figure 7:**
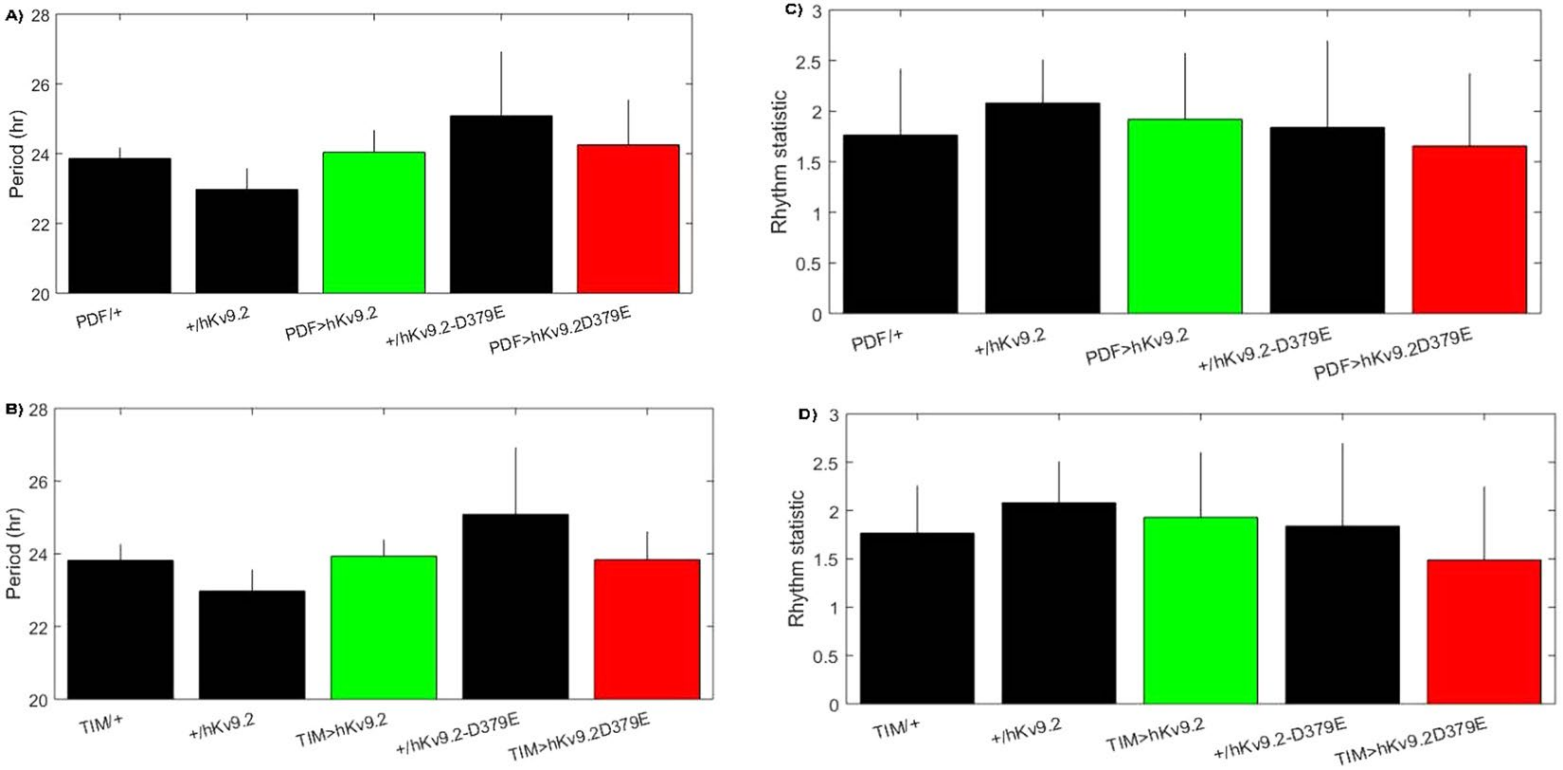
Effects of Expression of hKv9.2 and hKv9.2-D379E channels on Period or Rhythm Statistic. (**A**) Period of circadian locomotor rhythms in control flies and those expressing hKv9.2 or hKv9.2-D379E under the PDF promoter. (**B**) Period of circadian locomotor rhythms in control flies and those expressing hKv9.2 or hKv9.2-D379E under the TIM promoter. (**C**) The corresponding rhythm statistic obtained for flies using the PDF promoter. (**D**) The corresponding rhythm statistic obtained for flies using the TIM promoter.

**Figure 8:**
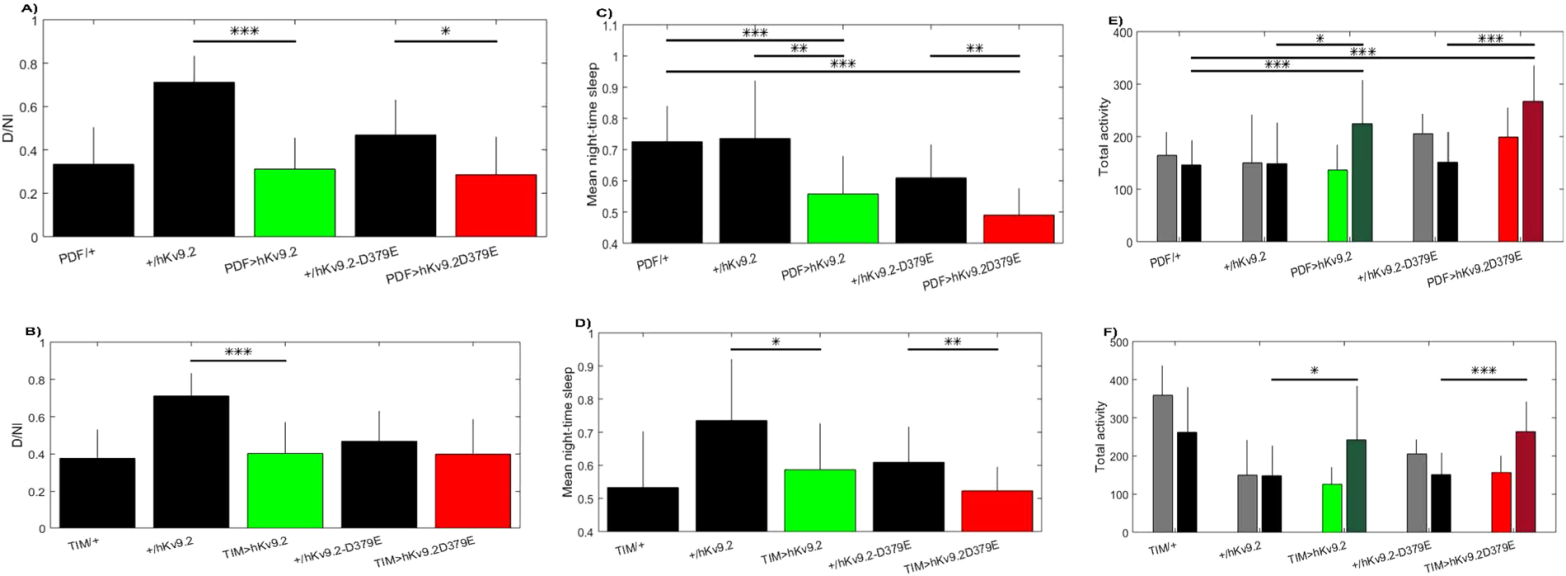
Effects of Expression of hKv9.2 and hKv9.2-D379E channels on diurnal/nocturnal index, average night time sleep and total night time activity. (**Top row**) Flies expressing hKv9.2 or hKv9.2-D379E under the PDF promoter and controls. (**Bottom row**) Flies expressing hKv9.2 or hKv9.2-D379E under the TIM promoter and controls. (**A**,**B**) The diurnal/nocturnal index (D/NI) examining the distribution of day-time and night-time activity. (**C**,**D**) The proportion of time flies spent asleep during the night-time, as defined as 5 minutes or more of inactivity. (**E**,**F**) The raw activity counts for flies split between day-time (lighter color, left-hand bars) and night-time (darker color, right-hand bars). Significant differences are observed in various comparisons summarized in Table 1.

While rhythmicity was unaffected by either hKv9.2 and hKv9.2-D379E channels (Table 1, Fig. 7), night-time activity was significantly increased (*p* = 0.02–0.0002), and concurrently night-time sleep was reduced (p = 0.02–0.0001) (Table 1, Fig. 8). Additionally, we observed a trend towards differences between hKv9.2 and hKv9.2-D379E channels (e.g. night-time sleep PDF > hKv9.2 vs PDF > hKv9.2-D379E p = 0.0745 and night-time activity PDF > hKv9.2 vs PDF > hKv9.2-D379E p = 0.0913), however the data did not reach statistical significance (Table 1). In sum, in addition to locomotor and electrophysiological defects, sleep is also disrupted by expression of hKv9.2 and hKv9.2-D379E channels in the *Drosophila* brain.

**Table 1:** Night-time activity and sleep activity in flies expressing hKv9.2 and hKv9.2-D379E.

Control	Treatment	Assay	Control	Treatment	*P*-value
PDF	PDF-hKv9.2	Night-time sleep	0.7253 ± 0.0224	0.5576 ± 0.0239	<0.0001
hKv9.2	PDF-hKv9.2	Night-time sleep	0.7352 ± 0.0464	0.5576 ± 0.0239	0.0025
PDF	PDF-hKv9.2-D379E	Night-time sleep	0.7253 ± 0.0224	0.4901 ± 0.0271	<0.0001
hKv9.2-D379E	PDF-hKv9.2-D379E	Night-time sleep	0.6092 ± 0.0220	0.4901 ± 0.0271	0.0026
hKv9.2	TIM-hKv9.2	Night-time sleep	0.7352 ± 0.0464	0.5869 ± 0.0361	0.0178
hKv9.2-D379E	TIM-hKv9.2-D379E	Night-time sleep	0.6092 ± 0.0220	0.5228 ± 0.0202	0.0066
PDF	PDF-hKv9.2	Night-time activity	146.1510 ± 11.6796	224.4103 ± 16.3261	0.00036
hKv9.2	PDF-hKv9.2	Night-time activity	148.3000 ± 24.7850	224.4103 ± 16.3261	0.0199
PDF	PDF-hKv9.2-D379E	Night-time activity	146.1510 ± 11.6796	267.0952 ± 18.2757	<0.0001
hKv9.2-D379E	PDF-hKv9.2-D379E	Night-time activity	151.1019 ± 19.2544	267.0952 ± 18.2757	0.00032
hKv9.2	TIM-hKv9.2	Night-time activity	148.3000 ± 24.7850	242.0729 ± 35.4191	0.0403
hKv9.2-D379E	TIM-hKv9.2-D379E	Night-time activity	151.1019 ± 19.2544	263.7882 ± 16.1640	0.00023
hKv9.2	PDF-hKv9.2	Diurnal/nocturnal index	0.7116 ± 0.0387	0.3115 ± 0.0284	<0.0001
hKv9.2-D379E	PDF-hKv9.2-D379E	Diurnal/nocturnal index	0.4685 ± 0.0542	0.2861 ± 0.0466	0.02
hKv9.2	TIM-hKv9.2	Diurnal/nocturnal index	0.7116 ± 0.0387	0.4033 ± 0.0433	<0.0001
PDF-hKv9.2	PDF-hKv9.2-D379E	Night-time sleep	0.5576 ± 0.0239	0.4901 ± 0.0271	0.0745
PDF-hKv9.2	PDF-hKv9.2-D379E	Night-time activity	224.4103 ± 16.3261	267.0952 ± 18.2757	0.0913

Comparison of the analyzed data gathered in the *Drosophila* Activity Monitor (DAM). The comparison shows the data ± S.E.M. for each comparison (summarized in Figs 7 and 8) of night-time activity, night-time sleep, or the diurnal/nocturnal index.

## Discussion

We have created a model of ET by expressing the human Kv9.2 channel subunit in *Drosophila*. We show that the hKv9.2 subunit can modulate the endogenous Shab (Kv2 subfamily) channel activity. Behavioral manifestations of nervous system dysfunction consistent with a hyperexcitable phenotype were observed in flies expressing the hKv9.2 or hKv9.2-D379E subunit during development and in adult neurons only. Studying the effects of hKv9.2 and hKv9.2-D379E on CNS neuronal activity, we showed that the mutant hKv9.2-D379E subunit showed significantly higher levels of inactivation than the wild-type hKv9.2 subunit and a significantly higher frequency of spontaneous firing rate of action potentials consistent with neuronal hyperexcitibility. A biophysical model of the electrical activity of the cell, combining the individual ionic currents in flies expressing hKv9.2 subunits, was in agreement with experimental data. Characterization of circadian behavior in flies expressing hKv9.2 and hKv9.2-D379E channels showed that while rhythmicity was unaffected significant differences were observed for night time activity and night time sleep. This is consistent with previous studies where hyperexcitation of lateral ventral neurons by NaChBac (low-threshold voltage-gated sodium channel for hyperexcitation of the cell) caused an increase in night activity and decrease in night sleep^34^ as seen in *Drosophila* models of Parkinson’s disease^35^. While effects on period circadian protein have been seen^36^, no differences were seen in the current study.

While we recognise that there are limitations to the *Drosophila* model that we have developed because of the lack of a clear *Drosophila* ortholog for Kv9.2, our data is consistent with previous studies that have demonstrated that Kv9.2 modulates the activity of Kv2 channels^2^ (such as *Drosophila* Shab), as well as observing significant differences in the phenotype (inactivation and spontaneous firing rate) between flies expressing the wild type and mutant hKv9.2 channels.

The pathogenicity of the *KCNS2* mutation (p.D379E) that we identified in an early-onset ET family^2^, that is the focus of the current study, is further supported by functional data from the *Drosophila* model and is consistent with previous observations reported by us including co-segregation with ET in a family, prediction as a pathogenic amino acid substitution by several variant prediction tools, absence from population databases, and its location in the pore domain of the Kv9.2 channel.

Our data is consistent with recent reports and observations that ET may represent a family of disorders of neurological channelopathies, with mutations identified in a voltage-gated K^+^ channel α subunit (the focus of this study) in a family with pure ET^2^, in voltage-gated sodium channel α subunits in a family with epilepsy and ET (*SCN4A*)^37^ and a family with familial episodic pain and ET (*SCN11A*)^38^. Further, the T-type calcium channel, Ca_v_3, has been implicated in neuronal autorhythmicity^39,40^ and is thought to underlie tremors seen in Parkinson’s disease^41^, enhanced physiological tremor, and in ET^42^ and T-type calcium channel antagonists have been shown to reduce tremor in mouse models of ET^43,44^. Nonetheless, the complete genetic basis for ET remains incomplete. Given the clinical and genetic heterogeneity observed in ET, further evaluation of ion channels as candidate genes for ET is warranted.

## Methods

### Transgenic *Drosophila*

Human *Kv9.2* sequenced-verified cDNA was obtained from the Mammalian Gene Collection (Clone ID: 5199736)(GE Dharmacon, Lafayette, Co). The RNA source for the cDNA was from an anonymous pool of 6 male brains, age range 23–27 years old. The library was oligo-dT primed and directionally cloned into *pCMV-SPORT6* vector. Human *Kv9.2* was transferred and cloned from the *pCMV-SPORT6* vector by TOPO® cloning (Thermofisher scientific, Waltham, MA) into the *pBID-UAS Drosophila* vector^45^.

Site directed mutagenesis was used to generate mutant human *Kv9.2*, c.1137 T > A, (p.D379E) using the Quick-change II site directed mutagenesis kit (Agilent Technologies, Santa Clara, CA) from the human *Kv9.2* sequenced-verified cDNA. Germ-line transformants were generated with PhiC31 integrase with Chromosome II attP40 landing site. *Drosophila* were maintained with standard conditions and food. *Uas-hKv9.2* and *uas-hKv9.2-D379E* were crossed to *Elav(c155)-Gal4* stock (Bloomington stock number-458) for pan-neural expression and *pdf-Gal4*, *pdf-rfp* for electrophysiological characterization^29,30^. Post-developmental effects utilized GAL80^TS^ ro, restricting expression to adult neurons^46–48^. For electrophysiological and circadian analysis, expression was restricted to the LNV clock neurons by use of pdf-Gal4 or throughout the clock system through tim-Gal4.

### Western Blot Analysis

An antibody to human Kv9.2 (Rabbit anti-Kv9.2, Thermofisher PA5-29511) was used for Western blot analysis. The Kv9.2 antibody recombinant fragment corresponds to a region within amino acids 175–470 of human Kv9.2.

### Negative Geotaxis Climbing Assay

The loss of climbing response was used to monitor ageing-related locomotor changes in *Drosophila*^14,49^. The climbing assay was performed as previously described^14,49^. We assessed 20 flies per vial for each transgenic and control line. Five trials were conducted for each vial. The average climbing rate was determined by measuring the time for the first fly to climb 17.5 cm. Climbing response was assessed at the following time points: Day 7, 14, 21, 28, 35, 42, 49, and 56.

#### Expression of hKv9.2 and hKv9.2-D379E channels in adult neurons after development and effects on lifespan

The climbing assay was performed as described for transgenic and control lines except 10 flies per vial were assessed for each line.

### Lifespan Assay

Lifespan assays were performed as described previously^50^. Briefly, 200 virgin female flies per line were sex-segregated within 4 h of eclosion and maintained in small laboratory vials (n = 20 per vial) containing fresh food in a low-temperature incubator at 25 °C and 40% humidity on a 12/12 h dark/light cycle. The flies were then transferred to fresh food vials every 2-3 days and mortality recorded.

#### Expression of hKv9.2 and hKv9.2-D379E channels in adult neurons after development and effects on lifespan

A total of 100 flies per line were sex-segregated within 4 h of eclosion and age-matched flies were maintained in small laboratory vials (n = 10 per vial) containing fresh food in a high-temperature incubator at 29 °C and 40% humidity on a 12/12 h dark/light cycle. The flies were then transferred to fresh food vials every 2-3 days and mortality recorded.

### Anesthetization Induced Leg Shaking and Wing Scissoring Behavior

Adult flies were anesthetized with ether and examined for leg shaking and wing scissoring as described previously^23,24^.

### Abnormal Wing Posture and Behavior Analyses

For abnormal wing posture analysis, flies were aged as in the lifespan assays. The abnormal wing posture penetrance was calculated as the percentage of flies with elevated or drooped wing posture. For each experiment, at least 10 flies were scored for their wing posture phenotype for each genotype.

### Electrophysiology

Experiments were conducted on wild type control: *pdf-Gal4; pdf-rfp*, as well as the two experimental lines: *pdf-Gal4* > *uas-hKv9.2; pdf-rfp* and *pdf-Gal4/uas-hKv9.2-D379E; pdf-rfp*. Flies were housed in 12 h light: 12 h dark cycles to facilitate recordings throughout the day. All flies were raised at 25 °C (incubators) at humidity of 60%.

Whole fly brains were dissected from CO_2_ anaesthetized adult flies aged between 0–5 days post-eclosion just before patch clamping using a previously established protocols^29,30^. Dissections were conducted in standard *Drosophila* external solution. The brain was then transferred to a recording chamber and held in place using a brain harp.

Whole-cell and patch-clamp recordings were made from the red fluorescent protein (RFP)-tagged pigment dispersing factor (PDF)-positive large Lateral Neuron ventral (l-LNv) clock neurons using a Multiclamp 700B amplifier and Axon Digidata 1440A digitizer in a recording chamber filled with *Drosophila* external solution as described previously^29,30^. Glass pipettes (8–15 MΩ) were pulled using a Sutter P-1000 puller and filled with internal solution. The resulting signal was then monitored and recorded using pClamp10 Clampex software and pipette offsets were zeroed prior to cell contact with the pipette capacitance being compensated upon contact. Subsequent signals were passed through a 10 kHz low-pass Bessel filter and sampled at 20 kHz. The cell-attached configuration was established by gentle suction applied through the pipette holder, and subsequent whole-cell configurations utilized a stronger pulse of negative pressure to break into the cell through the membrane.

The standard *Drosophila* external solution consisted of (in mM) 101 NaCl, 1 CaCl_2_, 4 MgCl_2_, 3 KCl, 5 glucose, 1.25 NaH_2_PO_4_, and 20.7 NaHCO_3_ with pH 7.2 and osmolality 250 mOsm. The internal solution consisted of (in mM) 102 K-gluconate, 0.085 CaCl_2_, 1.7 MgCl_2_, 17 NaCl, 0.94 EGTA, and 8.5 HEPES with pH 7.2 and osmolality 235 mOsm. The pH was increased with NaOH for the external solution and KOH for the internal solution and decreased with HCl for both. Stock solutions of guangxitoxin-1E (GxTX, Alomone labs) were made up using external solution. The resulting junction potential for these solutions has been calculated as being −13 mV (data shown has been adjusted to account for this). During the recordings, the drug solutions are added by pipette to 1 ml of external solution in the recording chamber to achieve the final drug concentration.

Voltage-clamp recordings were initially held at a membrane potential of −93 mV. For I-V relationships, a standardized protocol was used consisting of a hyperpolarizing step of −40 mV to −133 mV for 500 ms and then steps in increments of 10 mV from −93 mV to −3 mV for a duration of 200 ms before a return to the holding potential of −80 mV. To measure Shab currents, voltage-clamp recordings were performed before and after application of 10 nM GxTX^51^, the difference between the two conditions gives the subtractive Shab current. Wash-out of the drug recovered the channel current (88.64% ± 3.06%).

### *Drosophila* Activity Monitor (DAM)

Monitoring of the circadian activity of *Drosophila* was performed by measuring the locomotion of individual flies in a *Drosophila* activity monitor (DAM, Trikinetics Inc USA) system^52^. Male flies were initially held in 5 days of 12:12 LD (light/dark) before release into 7 days of DD (constant darkness). Analysis of activity was performed in Matlab and tubes showing no activity or early death were excluded from the analysis.

### Computational modeling

Computational models of the Shab channel ionic current, with addition of hKv9.2 or hKv9.2-D379E channels were generated by a nonlinear optimization algorithm fitting the electrophysiological data to Hodgkin-Huxley equations^53^ of the form:

1 $$I=gmax\times ({m}^{P}\,\ast \,{h}^{Q})\times (Vt-E)$$

where the current, *I*, depends on the maximal conductance (*gmax*), the reversal potential of the channel (*E*), the membrane voltage (*Vt*), and the activation and inactivation ion channel gating variables (*m* and *h*). The gating variables are given by the equations:

2 $$\frac{dm}{dt}=\frac{{m}_{\infty }-m}{{\tau }_{m}},$$

3 $${m}_{\infty }=\frac{1}{1+\,{e}^{-\frac{V-Vh}{k}}},$$

4 $${\tau }_{m}=Amp\times {e}^{-\frac{V-Vmax}{\sigma }}$$

5 $$\frac{dh}{dt}=\frac{{h}_{\infty }-h}{{\tau }_{h}},$$

6 $${h}_{\infty }=\frac{1}{1+\,{e}^{\frac{V-Vh}{k}}},$$

7 $${\tau }_{h}=Amp\times {e}^{-\frac{V-Vmax}{\sigma }}$$

The individual ionic current models fitted to our data were then combined in a model of the electrical behavior of the whole cell. The resulting whole-cell computational model builds upon a previous model of suprachiasmatic nucleus (SCN) clock neurons^54^ which incorporates Na^+^, K^+^, Ca^2+^, and leak currents. However, in our model the original composite K^+^ current is separated into the four currents mediated via the major voltage-gated K^+^ channels (Shaker Kv1, Shab Kv2, Shaw Kv3 and Shal Kv4) found in the l-LNvs based on electrophysiological recordings; while Kv2 refers to either the native Shab current, native Shab with human hKv9.2, or native Shab with hKv9.2-D379E. The current balance equation then is:

8 $$\begin{array}{c}C\frac{dV}{dt}={I}_{app}-{g}_{Na}{m}^{3}h(V-{E}_{Na})-{g}_{Ca}mh(V-{E}_{Ca})-{g}_{K1}{m}^{4}h(V-{E}_{K})\\ \,\,\,\,-{g}_{K2}{m}^{4}h(V-{E}_{K})-{g}_{K4}{m}^{4}h(V-{E}_{K})-{g}_{K3}{m}^{4}h(V-{E}_{K})-{g}_{leak}(V-{E}_{leak})\end{array}$$

where *E*_*Na*_ = 52 mV, *E*_*Ca*_ = 132 mV, *E*_*K*_ = −90 mV, and *E*_*leak*_ = −7 mV are the reversal potentials. These were calculated using the Nernst and Goldman-Hodgkin-Katz voltage equations based on the internal and external solutions used.

### Statistical Analysis

Statistical analysis was carried out using the Prism 7.0 (GraphPad Software, Inc., La Jolla, CA) software. Analysis of climbing response during lifespan was performed using unpaired student’s t-test (one unpaired t-test per row) in Prism 7.0. Data were represented as the mean and standard error of the mean (S.E.M.) from at least ten independent experiments at each week. Comparisons included *Elav/*+ versus *Elav* > *hKv9.2*, *Elav*/+ versus *Elav* > *hKv9.2-D379E* and *Elav* > *hKv9.2* versus *Elav* > *hKv9.2-D379E*. The false discovery rate (FDR) approach method used was the two-stage step up method of Benjami, Krieger and Yekutiel (FDR (Q) = 1%). Comparison of survival curves was performed using the Log-rank (Mantel Cox) test and the Gehan-Breslow-Wilcoxon test in Prism 7.0. Analysis of electrophysiology data was performed using pClamp10 Clampfit software and Matlab (Mathworks).

### Data availability

The authors declare that all data supporting the findings of this study are available within this article. Supplementary information files are available from the corresponding authors upon reasonable request.

## Electronic supplementary material

Supplementary Information

Supplementary Video 1A

Supplementary Video 1B

Supplementary Video 2A

Supplementary Video 2B
