# Supplementary material for: A Drosophila Model of Essential Tremor
Source: Sci Rep. 2018 May 16;8:7664. doi: 10.1038/s41598-018-25949-w (PMC5955955; doi:10.1038/s41598-018-25949-w)
Supplement: Supplementary file 1 — Supplementary Information [file 41598_2018_25949_MOESM1_ESM.doc]

**A *Drosophila* Model of Essential Tremor**

Philip Smith, Ronald Arias, Shilpa Sonti, Zagaa Odgerel, Ismael Santa-Maria, Brian D. McCabe, Krasimira Tsaneva-Atanasova, Elan D. Louis, James J.L. Hodge and Lorraine N. Clark

**Supplementary Figures**


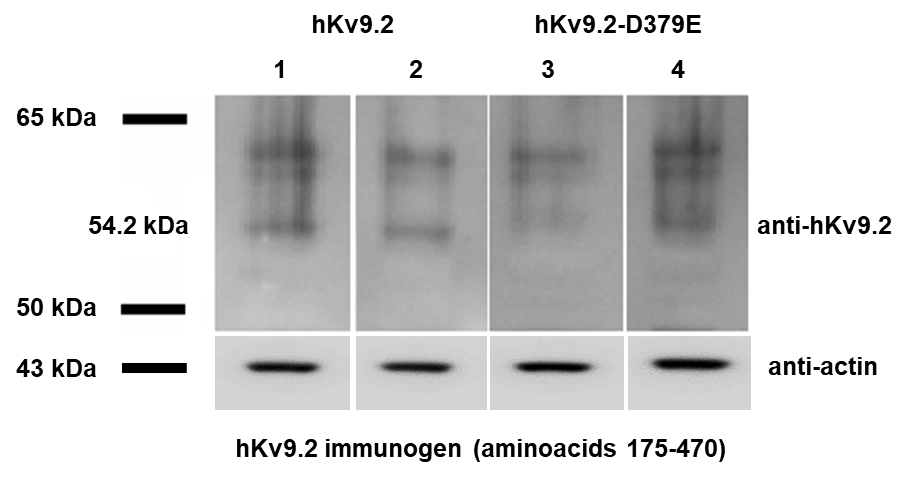


**Supplementary Figure 1. Western Blot Analysis of hKv9.2 in Transgenic *Drosophila* Lines.** Western blot analysis was used to verify transgenic expression of hKv9.2 and hKv9.2-D379E in *Drosophila*. Using a hKv9.2 immunogen corresponding to amino acids 175-470, we detected a 54.2KDa band corresponding to human hKv9.2. Lanes 1 and 2, technical replicates of wildtype hKv9.2. Lanes 3 and 4, technical replicates of mutant hKv9.2-D379E. A loading control detects a 43KDa band corresponding to β-actin indicating equivalent amounts of protein in lanes 1,2,4 and 5. Lanes were cropped from different parts of the same gel. The western blot analysis of hKv9.2 in transgenic *Drosophila* lines was repeated in three independent experiments. The full length Western blot is shown in Supplementary Figure 3.


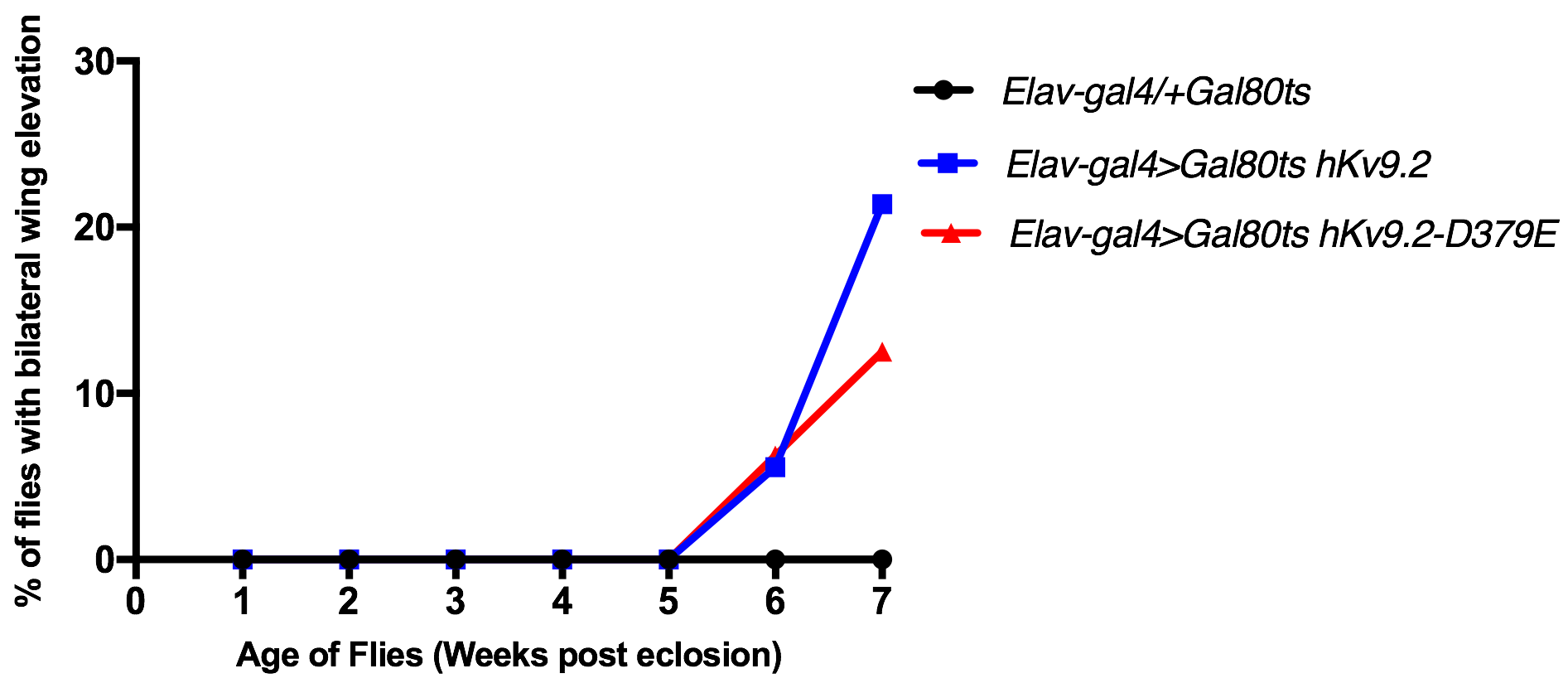


**Supplementary Figure 2. Wing posture and motility deficits in Flies expressing the wildtype or mutant hkv9.2 channel in post-metamorphic neurons.** A total of 20 flies per line were collected shortly after eclosion and age-matched flies were maintained in small laboratory vial (n=10 per vial) containing fresh food in a low-temperature incubator at 29°C and 40% humidity on a 12/12 h dark/light cycle. The flies were observed for a wing posture phenotype every week. Significant differences were observed between *Elav-gal4/+Gal80ts* and *Elav-gal4>Gal80ts* *hKv9.2* (*p*<0.0001) or *Elav-gal4/+Gal80ts* and *Elav-gal4>Gal80ts* *hKv9.2-D379E (p*<0.0001).


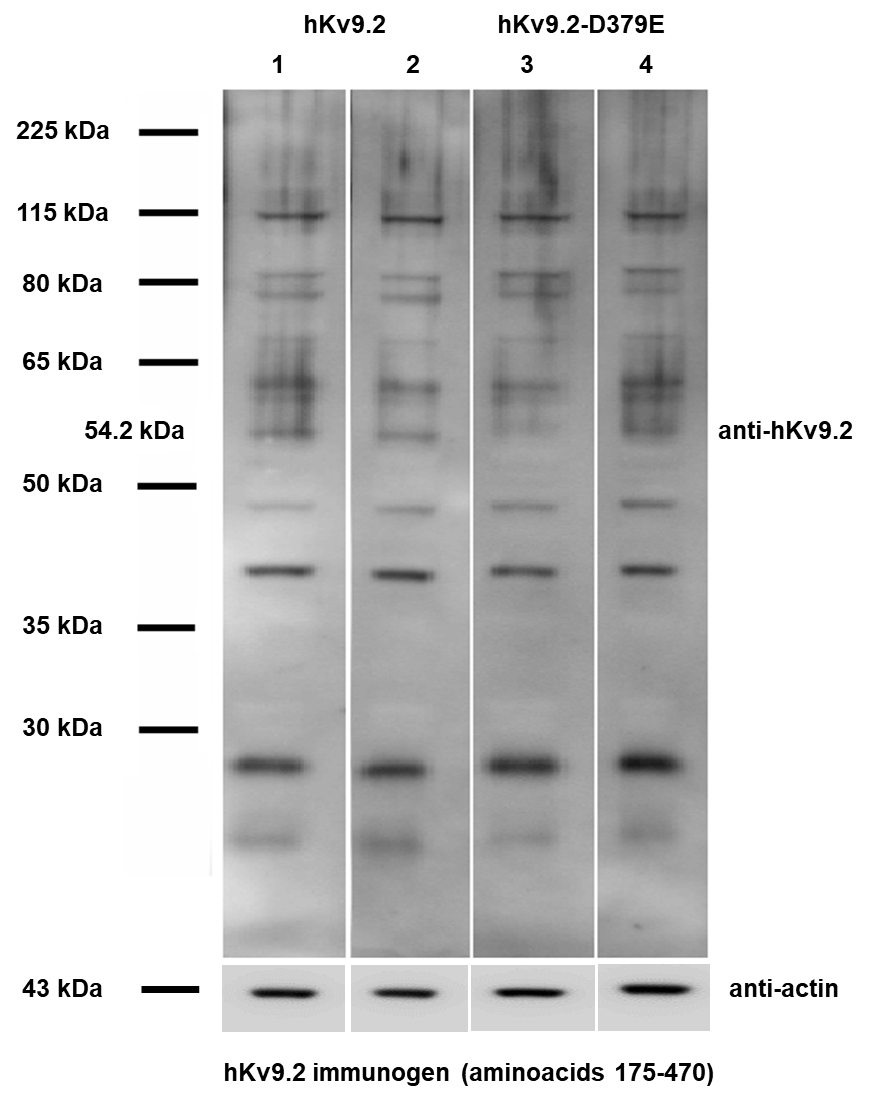


**Supplementary Figure 3. Western Blot Analysis of hKv9.2 in Transgenic *Drosophila* Lines. The full length Western blot corresponding to Supplementary Figure 1 is shown.** Western blot analysis was used to verify transgenic expression of hKv9.2 and hKv9.2-D379E in *Drosophila*. Using a hKv9.2 immunogen corresponding to amino acids 175-470, we detected a 54.2KDa band corresponding to human hKv9.2. Lanes 1 and 2, technical replicates of wildtype hKv9.2. Lanes 3 and 4, technical replicates of mutant hKv9.2-D379E. A loading control detects a 43KDa band corresponding to β-actin indicating equivalent amounts of protein in lanes 1,2,4 and 5. Lanes were cropped from different parts of the same gel. The western blot analysis of hKv9.2 in transgenic *Drosophila* lines was repeated in three independent experiments.

**Supplementary** **Videos**

**Supplementary Movie 1. Abnormal wing posture and bilateral elevated wing phenotype in hKv9.2 transgenic lines.**  Flies expressing A) hKv9.2 or B) hKv9.2-D379E pan-neuronally displayed an abnormal wing posture, with bilateral wing elevation, with onset 7-21 days post eclosion

**Supplementary Movie 2. Anesthetization induced shaking in adult flies expressing hKv9.2 or hKv9.2-D379E pan-neuronally.**

A) Adult flies expressing hKv9.2 or hKv9.2-D379E pan-neuronally showed leg shaking, abdominal pulsations and body shuddering under ether anesthetization consistent with the electrophysiology data that showed neuronal hyperexcitability. B) Etherised adult control flies do not display the abnormal leg shaking, abdominal pulsations and body shuddering observed in hKv9.2 transgenic lines.
